# Prevalence and determinants of health-related quality of life in chronic obstructive pulmonary disease patients in Yaoundé, Cameroon: a pilot study

**DOI:** 10.11604/pamj.2024.47.39.39701

**Published:** 2024-02-01

**Authors:** Abdou Wouoliyou Nsounfon, Massongo Massongo, Alain Kuaban, Marie Elisabeth Ngah Komo, Virginie Poka Mayap, Marie Christine Ekongolo, Eric Walter Pefura Yone

**Affiliations:** 1 Department of Internal Medicine and Specialties, Faculty of Medicine and Biomedical Sciences, University of Yaoundé I, Yaoundé, Cameroon,; 2 Internal Medicine and Specialties Unit, Central Hospital of Yaoundé, Yaoundé, Cameroon,; 3 Respiratory Medicine Unit, Jamot Hospital, Yaoundé, Cameroon,; 4 Infectious Diseases Unit, Limbé Regional Hospital, Limbé, Cameroon

**Keywords:** Health-related quality of life, chronic obstructive pulmonary disease, Saint George’s respiratory questionnaire, Cameroon

## Abstract

**Introduction:**

the present study aimed to assess the health-related quality of life (HRQL) and identify the factors associated with poor quality of life, among chronic obstructive pulmonary disease (COPD) patients.

**Methods:**

we conducted a cross-sectional study at Jamot Hospital and Polymere Medical Center, Yaoundé, from February 1 to June 30, 2020. All consent adult COPD patients who were followed in both centers during the recruitment period were included. The Saint George's Respiratory Questionnaire (SGRQ) was used to assess HRQL. Poor quality of life was defined by an SGRQ score ≥30. Data analysis was performed using IBM SPSS Statistics 23.0 (IBM Corp., Armonk, New York, USA) software. Multiple logistic regression was used to identify the factors associated with poor quality of life. The statistical significance threshold was set at 0.05.

**Results:**

of the 63 patients invited to participate in the study, only 29 were finally included. Almost 3/5 (58.6%) were males, and their median age (interquartile range, IQR) was 68.0 (57.0 - 74.5) years. The median HRQL score (IQR) was 44.2 (23.2 - 65.0). The prevalence (95% confidence interval, 95% CI) of poor HRQL was 65.5% (48.3 - 82.8) %. The history of exacerbations during the last 12 months [odds ratio (95% CI) = 12.3 (1.1 - 136.7); p=0.04] emerged as the sole independent predictor of poor HRQL.

**Conclusion:**

the prevalence of poor health-related quality of life was high in these COPD patients. The presence of exacerbations in the past 12 months was an independent factor associated with poor HRQL in patients with COPD.

## Introduction

Chronic obstructive pulmonary disease (COPD) is a common disease, affecting nearly 10% of the adult population worldwide [1]. It is a major public health problem because of its increasing morbidity and mortality, the cost of its management, and the impact on patients' quality of life [2-4]. It therefore constitutes a major economic and social burden that is constantly growing. Epidemiological studies have been conducted in Africa on COPD for a few years [5-8], but they remain insufficient according to the World Health Organization (WHO) [9]. In Cameroon, some studies carried out on COPD have found a prevalence varying between 2.4% and 5.2% [5,10-12]. These are essentially prevalence studies (with or without an etiological component) carried out in the community.

The natural history of COPD includes recurrent episodes of exacerbations that can lead to a loss of patient autonomy, making it the leading cause of chronic respiratory failure [13]. It is a debilitating disease and a frequent cause of respiratory disability, which is responsible for a deterioration in the quality of life of these patients. Indeed, 50% to 65% of COPD patients have poor health and quality of life [14-16]. Impaired quality of life has been shown to increase the risk of morbidity and mortality in COPD patients [17-19]. Hence, improving the quality of life is now one of the objectives of COPD management.

Before putting in place strategies targeting the improvement of quality of life in COPD patients, it seems appropriate to evaluate their characteristics. This could be done in local structures involved in the care of COPD patients in Yaoundé in a bid to ensure the development of strategies specific to the context. This study aimed to assess the quality of life of patients with chronic obstructive pulmonary disease (COPD) and identify factors associated with poor quality of life.

## Methods

**Study design and setting:** it was a cross-sectional study in two health facilities in the city of Yaoundé: Jamot Hospital (JHY) and Polymere Medical Center, conducted from February 1 to June 30, 2020. Yaoundé: Jamot Hospital is the national reference center for the management of respiratory and mental diseases. As such, it receives patients from all regions of Cameroon and the Central African subregion. Polymer Medical Center is a private health facility specializing in the exploration and management of respiratory and allergic diseases.

**Study population and sampling:** all consecutive adult COPD patients who were being followed in the study sites were invited to participate in the study. Only those patients who gave informed and written consent with no current or recent (less than 1 month) exacerbation were finally enrolled in the study. The study size was estimated using online OpenEpi, version 3. Assuming a 63.8% prevalence of poor quality of life among COPD found by Irianti in 2018 [9].

**Organization:** patients were selected from consultation registers. The first contact was made by telephone interview or at the end of a consultation with the referring pulmonologist. This made it possible to check the patient's eligibility, inform the patient about the study, invite him to participate, and give him/her the consent form. When conditions were permitted, the inclusion visit occurred during the second contact or immediately following the first one. It was done in a room reserved for the purpose away from prying eyes and surrounding noise, through a face-to-face interview with the patient, using a previously tested paper data collection form.

**Chronic obstructive pulmonary disease definition:** the most recent spirometry was required to make the diagnosis and severity of COPD. The presence of a post-bronchodilator FEV1/FVC ratio (forced expiratory volume in 1s/forced vital capacity) below the lower limit of normal (LLN) was used to confirm the presence of persistent airflow limitation and thus of COPD [20]. Chronic obstructive pulmonary disease severity was defined according to the 2006 Global Initiative for Obstructive Lung Disease (GOLD) classification [21]. It included 4 stages of severity based on the post-bronchodilator FEV1, expressed as a percentage of the predicted value.

**Baseline data:** anamnestic, symptomatic, and personal data were collected during the interview. Respiratory symptoms were cough, expectoration, wheezing, hemoptysis, and dyspnea. The severity of dyspnea was assessed using the Modified Medical Research Council (mMRC) scale [22]. Concerning smoking habits, an "ex-smoker" was defined as a person who usually used tobacco and had stopped this for at least 6 months. A smoker was defined as a person who was still smoking or who had stopped for less than 6 months. A Non-smoker was defined as a person who had never smoked or had smoked an average of less than 1 cigarette per day for less than 1 year.

The diagnostic, therapeutic, and evolutionary data for COPD were collected from the patient's medical file. An exacerbation was defined as a worsening of respiratory symptoms requiring treatment with oral corticosteroids, an antibiotic, or both [23,24]. The patient was considered a frequent exacerbator if he had at least 2 exacerbations per year. Anthropometric (abdominal circumference, weight, height) and physical data were collected during the physical examination performed by the investigator. Transcutaneous oxygen saturation of hemoglobin (SpO_2_) (%) was measured using a pulse oximeter (OXY-ONE lite) placed at the level of the fingertip. Depressive symptoms and their level of severity were assessed using the "Patient Health Questionnaire-9 (PHQ-9). Patients were categorized according to their score as having depressive symptoms (5 or more) or not (less than 5) [25].

**Quality of life assessment:** the health quality of life (HRQL) was assessed using the specific respiratory questionnaire "St George´s Respiratory Questionnaire" (SGRQ) [26]. This questionnaire was developed and initially validated at St George's Medical Hospital in London in asthmatic patients [26,27]. It has two parts, subdivided into three components. The first part corresponds to the symptoms component and comprises eight items. The second part includes the impact component (26 items distributed in sections 1, 3, 4, 5, and 7) and the activities component (16 items distributed in sections 2 and 6). Each item is assigned a unique weight (between 0 and 100) derived from previous data. The minimum weight is 0 and the highest is 100. For each component, the sum of the weights assigned to each response is calculated, and the score is obtained by dividing this sum by the maximum possible score and expressing the result as a percentage. The total score is obtained in the same way as the component scores, and this is done by using the three component scores. The total score is calculated by adding all responses obtained from the questionnaire and expressing the result as the percentage of the maximum possible for the entire questionnaire. Finally, the higher the SGRQ score, the lower the HRQL for the patient. The threshold used to define a poor HRQL was 30 [28]. It is this usual threshold that we used in our study.

**Data analysis:** data were recorded using CSPro7.0 software (United States Census Bureau, Washington, USA) and analyzed using IBM SPSS 23.0 software (IBM Corp, Armonk, New York, USA). Quantitative data were presented using the median and the interquartile range (IQR). Univariate logistic regression was used to search for associated factors with poor HRQL. The explanatory variables associated with poor HRQL with a p-value < 0.10 were selected for the multivariate analysis. Multivariable logistic regression was used to identify independent determinants of poor HRQL in patients with COPD. The strength of the association was determined by the adjusted odds ratio (aOR) and its 95% confidence interval (95% CI). The simplification of the models was carried out according to the stepwise regression method. At each step, the variable with the highest p-value was extracted from the model, provided that its removal did not lead to a variation in OR greater than 30%. In this case, the variable concerned was kept in the model and considered as a confounding factor. The simplification stopped when all variables in the model had a p-value < 0.05 or there were still variables with a p-value ≥0.05 whose removal led to a significant modification of the aOR of another variable.

**Ethics:** this study was approved by the ethics committee of the Faculty of Medicine and Biomedical Sciences of the University of Yaoundé I. It received administrative authorization from Yaoundé Jamot Hospital and the Polymere Medical Center. The St George´s University of London (St George´s Hospital Medical School) permitted to use of the Saint George Questionnaire (SGRQ) in this study. Participants´ data were collected and analyzed anonymously.

## Results

**Figure 1:**
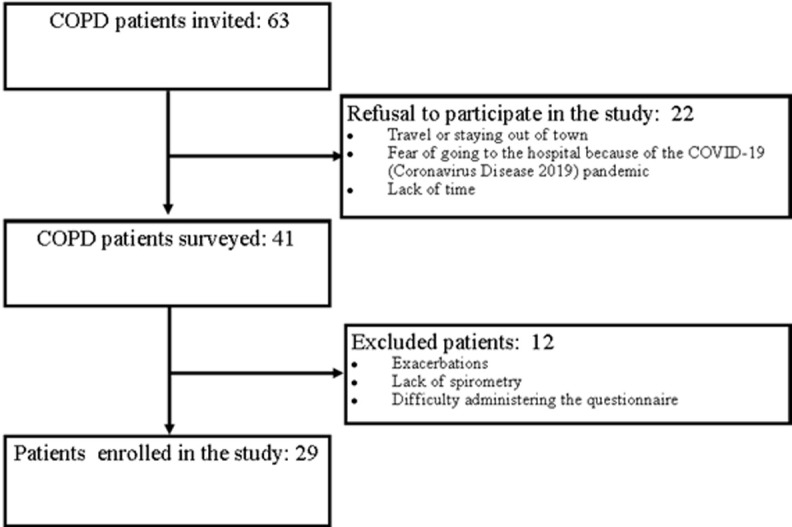
flow-chart of the selection of participants

Sixty-three COPD patients were invited to participate in the study. Twenty-two patients declined the invitation, 12 were excluded (for exacerbation, lack of spirometry, or difficulty in administering the questionnaire) and 29 finally entered the study (Figure 1).

**Baseline characteristics of the study population:** of these 29 patients, 17 (58.6%) were males and the median age (interquartile range, IQR) was 68.0 (57.0 - 74.5) years, with extreme ages of 34 and 88 years. Almost half of the participants (48.3%) were married, and more than half of them had no professional activity (55.2%). The median (IQR) time of discovery of COPD was 1.00 (0.1 - 3.50) years. Stage III COPD (44.8%) was the most frequent. The most commonly known risk factor for COPD was tobacco (37.9%), and 44.8% of patients had no identified risk factors. Seventeen patients (58.60%) had experienced at least one exacerbation of COPD in the last 12 months. The most frequent comorbidity was arterial hypertension (20.7%). Slightly less than half of the patients were smokers or ex-smokers. Dyspnea (69.0%) and cough (62.1%) were the most common respiratory symptoms. Stage I dyspnea was the most frequent (50.0%). Depressive symptoms based on PHQ9 were present in 15 (51.7%) patients. These data are shown in Table 1. The median (IQR) body mass index and transcutaneous oxygen saturation were 24.8 (20.9 - 29.0) kg/m^2^ and 95.0 (92.0 - 97.0)% respectively.

**Table 1:** general characteristics of COPD patients, February- June 2020, Yaoundé, N=29

Variables		Number (N=29)	Percentage (%)
**Sex**	Male	17	58.6
	Female	12	41.4
**Marital status**	Single	4	13.8
	Married	14	48.3
	Widow/widower	9	31.0
	Divorced	2	6.9
**Profession**	No activity	16	55.2
	Activity	13	44.8
**Stage of COPD**	Stage I	4	13.8
	Stage II	8	27.6
	Stage III	13	44.8
	Stage IV	4	13.8
**Known risk factors for COPD**	Tobacco smoke	11	37.9
	Biomass	1	3.4
	Sequelae of tuberculosis	3	10.3
	Professional exposure	1	3.4
	Unknown	13	44.8
**Treatment of COPD**	Yes	24	82.8
	No	5	17.2
**Frequency of exacerbations during the last 12 months**	0	12	41.4
	1	7	24.1
	≥ 2	10	34.5
**Comorbidities**	Arterial hypertension	6	20.7
	Obesity	5	17.2
	Diabetes	2	6.9
	HIV	4	13.8
	Heart failure	4	13.8
**Consumption of tobacco**	Never	15	51.7
	Former smoker	2	41.4
	Active smoker	2	6.9
**Current respiratory symptoms**	Cough	18	62.1
	Sputum production	15	51.7
	Dyspnea	20	69.0
**Stages of dyspnea (mMRC) (N=20)**	Stage I	10	50.0
	Stage II	4	20.0
	Stages III	2	10.0
	Stage IV	4	20.0
**Depressive symptoms**	Absent	14	48.3
	Present	15	51.7

mMRC: modified Medical Research Council; COPD: chronic obstructive pulmonary disease

**Quality of life score and prevalence of poor health-related quality of life:** the median (IQR) global HRQL score of COPD patients was 44.24 (23.17 - 64.97). Domains´ median scores are present. The symptom, activity, and impact domains scores (IQR) were 47.5 (31.5 - 64.6), 53.5 (24.1- 79.1), and 35.1 (18.5 - 57.4), respectively. Nineteen patients had an SGRQ score ≥30, giving a poor health-related quality of life prevalence (95% CI) in COPD patients of 65.50% (48.30% - 82.8%).

**Predictors of poor health-related quality of life:** in univariate analysis, advanced stages of COPD (GOLD III and IV) [OR (95% CI) = 6.53 (1.20- 35.57); p=0.03], the presence of dyspnea [OR (95% CI) = 8 (1.37 - 46.81); p=0.021] and exacerbations in the last 12 months [OR (95% CI) = 6.53 (1.20 - 35.57); p=0.03] were associated with poor health-related quality of life in COPD patients (Table 2). During the simplification of the model and after adjusting for the two confounding factors (COPD stage and history of dyspnea), only a history of exacerbation in the last 12 months [aOR (95% CI) = 12.34 (1.12 - 136.69); p=0.041] was independently associated with poor health-related quality of life (Table 3).

**Table 2:** univariate analysis of the association between quality of life and clinical data of COPD, February - June 2020, Yaoundé, N=29

Variables	Poor quality of life, n (%)	OR	95% IC	P-value
Ages, in years		0.94	0.89 - 1.01	0.067
Stages of COPD				
Stages I and II	5 (41.7)	1		
Stages III and IV	14 (82.4)	6.53	1.20 - 35.57	0.030
Exacerbations in the last 12 months				
Yes	14 (82.4)	6.53	1.20 - 35.57	0.030
No	5 (41.7)	1		
Dyspnea				
Yes	16 (80.8)	8.00	1.37 - 46.81	0.021
No	3 (33.3)	1		
Profession				
No activity	13 (81.3)	5.06	0.96 - 26.66	0.056
Activity	6 (46.2)	1		

**Table 3:** representation of the factors independently associated with poor quality of COPD patients in multivariate analysis, N=29

Variables	Initial model		Final model	
	OR (95% CI)	P-value	aOR (95% CI)	P-value
Ages in years	1.01 (0.93 - 1.10)	0.846		
Stages of COPD (GOLD)				
I and II	1		1	
III and IV	3.51 (0.34 - 36.03)	0.290	4.61 (0.57 - 37.30)	0.152
Exacerbations in the last 12 months				
Yes	10.58 (0.83 - 134.29)	0.069	12.34 (1.12 - 136.69)	0.041
No	1		1	
Dyspnea				
Yes	10.51 (0.88 - 125.37)	0.063	10.71 (0.92 - 124.92)	0.059
No	1		1	
Profession				
No activity	1.92 (0.20 - 18.45)	0.574		
Activity	1			

GOLD: global initiative for chronic obstructive lung disease; COPD: chronic obstructive pulmonary disease; OR: odds ratio; aOR: adjusted odds ratio; CI: confidence interval

## Discussion

Our study aimed to assess the quality of life of patients with chronic obstructive pulmonary disease (COPD) in two healthcare facilities in Yaoundé (Yaoundé Jamot Hospital and the Polymere Medical Center) and to identify the predictive factors of poor health-related quality of life in these patients. The prevalence (95% confidence interval) of poor health-related quality of life (HRQL) in COPD patients was 65.5% (48.3% - 82.8%). The mean (SD) and median (IQR) HRQL scores were 44.78 (23.86) and 44.24 (23.17 - 64.97), respectively. Factors associated with poor quality of life were: advanced stage (GOLD III and IV) of COPD (OR = 6.53), presence of dyspnea (OR = 8), and exacerbations in the last 12 months (OR = 6.53). After adjusting for the confounding factors (severity, stage of COPD, and dyspnea), the history of COPD exacerbation in the last 12 months (OR = 12.34) was an independent factor associated with poor HRQL in patients with COPD.

The HRQL score of patients with COPD varies from one country to another and according to the socioeconomic conditions and the quality of care these patients receive. Ours was close to the total SGRQ score of 45 found in a multicenter study carried out in seven European countries by Jones *et al*. [29]. This similarity could reflect that, beyond the socio-economic conditions as well as the technical expertise and healthcare systems available, there are other factors inherent to patients that would seem to play a non-negligible role in the health-related quality of life of the latter, namely age, gender, disease duration, disease severity, and comorbidities [16]. In another multicenter study including European countries and the United States of America carried out by Carone *et al*. [18] the mean SGRQ score was 53, higher than ours. This difference in scores could be explained, among other things, by the study population. Indeed, our study included all patients with COPD who had not had recent exacerbations, whereas Carone *et al*. only included patients with COPD already at the stage of chronic respiratory failure and on mechanical ventilation. This could explain the higher score (and therefore a more impaired quality of life) in their study, compared to ours. A study was carried out at the Douala General Hospital in Cameroon by Ngahane *et al*. [30], and we found a mean SGRQ score of 53.1, which is higher than ours. Several factors known to alter the quality of life were present at higher values in this study when compared to ours, namely, the mean age (67.5 years vs. 64.7 years), the severity of COPD (stages III and IV: 68% vs 58.6%) and respiratory symptoms (cough: 87.8% vs 62.1%; dyspnea: 97.6% vs 69%). This could therefore explain the more impaired quality of life in this study when compared to ours.

The prevalence of poor quality of life in patients with COPD varies little. Ours was close to 64% and 65% found, respectively, by Iranti *et al*. in Indonesia [15] and Lee *et al*. in Korea [16]. Many factors influence the quality of life of patients with COPD. These include age, tobacco consumption, unfavorable socioeconomic conditions, nutritional status, recurrence of exacerbations, severity of COPD, depression, and the presence of respiratory symptoms (mainly dyspnea). Numerous studies have demonstrated the association between the severity of COPD and poor quality of life. Lee *et al*. [16] in Korea found a greater proportion of poor quality of life in patients with COPD at stages III and IV of GOLD compared to that of patients at stages I and II (80.4% vs. 59.2%). This same study found a significant association between the percentage (% predicted) of post-bronchodilator FEV1 and the SGRQ score in patients with mild to moderate COPD (r = -0.10; p = 0.002) than in severe to very severe patients (r= -0.22; p <0.001). Jones *et al*. in Europe also found that the deterioration of the state of health was associated with the severity of the obstruction of the airways and therefore with the level of severity of COPD [29].

In our study, the presence of dyspnea was associated with a poor quality of life in patients with COPD. This result is similar to those found in the literature [31-34]. Indeed, the Korean study by Lee *et al*. [16] found that dyspnea was independently associated with poor quality of life in patients with COPD regardless of the stage of severity of the disease (aOR: 4 - 5; p < 0.01). It was also shown in this study that the high proportion of patients with mild to moderate COPD having a poor quality of life (60%) despite relatively good lung function was mainly related to the presence of respiratory symptoms, especially dyspnea [16]. Indeed, although dyspnea was more common in patients with significant levels of bronchial obstruction, disability due to shortness of breath was common even in patients with stage I and II COPD, as found in a European study [30]. The study by Justine M *et al*. [32] showed a significant association between quality of life, pulmonary function, and dyspnea in patients with stable COPD. The dyspnea score was significantly correlated with the different components of the health-related quality of life score (p < 0.05). A multiple regression analysis indicated that the assessment of dyspnea was the most important predictor of the health status of patients with COPD [32].

Sanchez *et al*. [35] in their study conducted in Brazil on eighty-nine patients with COPD showed that the Modified Medical Research Council (mMRC) scale for evaluating dyspnea was a predictive factor of the SGRQ score (p < 0.001) and therefore the quality of life. A few studies have also found that, in addition to dyspnea, cough, and sputum, although less severe, were also associated with poor quality of life in patients with COPD regardless of the level of severity of bronchial obstruction (p < 0.01) [16]. All these results sufficiently show that the evaluation of respiratory symptoms and dyspnea in particular is an important factor in the prediction of health-related quality of life (HRQL) in patients with COPD and that the stage of severity of dyspnea influences quality of life more than the physiological measure of respiratory function. The burden of these significant respiratory symptoms in COPD, especially in cases of severe bronchial obstruction, reduces physical and psychological functioning, ultimately decreasing health-related quality of life [36]. Thus, the search for dyspnea and respiratory symptoms in general in a patient with COPD, even at an early stage or in a stable state, makes it possible to detect an alteration in the quality of life early and therefore improve management.

The natural history of COPD includes recurrent episodes of exacerbations, which are the most common reason for consultation, hospitalization, and death in these patients. Several studies have shown that these exacerbations are associated with a significant deterioration in the state of health of subjects with COPD and therefore have a definite impact on the health of patients [23,34,37,38]. In our study, we found that the presence of at least one exacerbation in the last 12 months is independently associated with poor quality of life in patients with COPD, a result similar to those of other studies [23,24,37]. In a prospective study on 156 subjects with COPD, Nishimura *et al*. [24] showed that 31% of these patients had experienced one or more exacerbations during 6 months of follow-up. Patients with exacerbations had a significant drop in SGRQ symptom scores (p<0.05), although there was no noticeable change in respiratory function. Seemungal *et al*. [37] reported that patients who had more than three exacerbations per year had significantly higher SGRQ scores and therefore a more impaired quality of life than those who had fewer. Results from the ISOLDE (Inhaled Steroids in Obstructive Lung Disease in Europe) trial of 613 patients with COPD showed that frequent exacerbations were independently associated with impaired quality of life (P< 0.0001) and a more rapid deterioration in the state of health (p=0.0003) [23]. Despite optimal management and rapid recovery from acute exacerbations, they have a significant and lasting effect on the state of health because convalescence is sometimes long even in the absence of additional exacerbations.

In addition, the delay in complete recovery in patients with frequent exacerbations may affect the further deterioration of the health status of these patients. This has been demonstrated in several studies [23,24,37]. Thus, exacerbations can have harmful and cumulative effects on the state of health. Therefore, to avoid further deterioration of the state of health, it is important to prevent recurrent exacerbations [23]. Our study did not reveal many other factors known to be associated with poor quality of life in patients with COPD, such as the low socioeconomic level [16], the presence of comorbidities [16,29], depression [14], tobacco consumption [39] and hypoxemia [40]. The reason for this could be the small size of our sample compared to other studies, which were mostly multicenter and included a large number of subjects.

The main limitations of our study were: i) cross-sectional nature, which does not allow a causal link to be established between the factors highlighted and the event of interest; ii) The subjective nature of quality-of-life assessment tools, the answers to which can lead to biases because they depend on the respondent's experience and susceptibility; iii) The small sample size, may mask certain associated factors. However, it is one of the pioneer studies in Cameroon that not only assessed the quality of life of patients with COPD but also assessed their depressive symptoms. This work is the first of its kind, which will make it possible to express to various actors in the health sector as well as to practitioners the burden of COPD on the health and the poor quality of life of the patients concerned. We hope that this study will allow better management of patients with COPD, which will no longer be limited to the prescription of drugs, but to a broad and multidisciplinary vision with under optimal conditions, the creation of a respiratory rehabilitation center in the major cities of Cameroon. We also hope to be able to continue it by extending it to other centers caring for patients with COPD, to improve its power and external validity.

## Conclusion

We found a high prevalence of poor health-related quality of life in patients with COPD. A factor independently associated with poor quality of life in COPD patients was the presence of exacerbations in the past 12 months. Thus, COPD constitutes a real burden on the state of health and the poor quality of life of the patients concerned. Holistic and multidisciplinary management should reduce the frequency of exacerbations and therefore improve the health of patients with COPD.

### What is known about this topic

- *Poor health-related quality of life (HRQL) is frequent among COPD patients;*
- *Poor quality of life is associated with COPD severity, dyspnea at baseline state, and the presence of exacerbations*.

### What this study adds

- *The prevalence of poor HRQL in our Cameroonian COPD patients was close to those recently found in Eastern settings;*
- *Predictors of poor HRQL in our patients were the same as described in the literature*.
